# A COVID-19 Supply Chain Management Strategy Based on Variable Production under Uncertain Environment Conditions

**DOI:** 10.3390/ijerph18041662

**Published:** 2021-02-09

**Authors:** Mohammed Alkahtani, Muhammad Omair, Qazi Salman Khalid, Ghulam Hussain, Imran Ahmad, Catalin Pruncu

**Affiliations:** 1 Industrial Engineering Department, College of Engineering, King Saud University, Riyadh 11421, Saudi Arabia; 2 Raytheon Chair for Systems Engineering (RCSE), Advanced Manufacturing Institute, King Saud University, Riyadh 11421, Saudi Arabia; 3 Department of Industrial Engineering, Jalozai Campus, University of Engineering and Technology, Peshawar 25000, Pakistan; muhamad.omair87@gmail.com (M.O.); qazisalman@uetpeshawar.edu.pk (Q.S.K.); 4 Faculty of Mechanical Engineering, GIK Institute of Engineering Sciences & Technology, Topi 23460, Pakistan; gh_ghumman@hotmail.com; 5 Department of Mechanical Engineering, Imperial College London, Exhibition Rd., London SW7 1AY, UK; 6 Design, Manufacturing & Engineering Management, University of Strathclyde, Glasgow G1 1XJ, UK

**Keywords:** risk supply chain management, COVID-19 pandemic, fuzzy costs, controllable production rate, demand depending emergency level, imperfect production

## Abstract

The management of a controllable production in the manufacturing system is essential to achieve viable advantages, particularly during emergency conditions. Disasters, either man-made or natural, affect production and supply chains negatively with perilous effects. On the other hand, flexibility and resilience to manage the perpetuated risks in a manufacturing system are vital for achieving a controllable production rate. Still, these performances are strongly dependent on the multi-criteria decision making in the working environment with the policies launched during the crisis. Undoubtedly, health stability in a society generates ripple effects in the supply chain due to high demand fluctuation, likewise due to the Coronavirus disease-2019 (COVID-19) pandemic. Incorporation of dependent demand factors to manage the risk from uncertainty during this pandemic has been a challenge to achieve a viable profit for the supply chain partners. A non-linear supply chain management model is developed with a controllable production rate to provide an economic benefit to the manufacturing firm in terms of the optimized total cost of production and to deal with the different situations under variable demand. The costs in the model are set as fuzzy to cope up with the uncertain conditions created by lasting pandemic. A numerical experiment is performed by utilizing the data set of the multi-stage manufacturing firm. The optimal results provide support for the industrial managers based on the proactive plan by the optimal utilization of the resources and controllable production rate to cope with the emergencies in a pandemic.

## 1. Introduction

Supply chain management (SCM) tends to organize and manage a complete set of activities integrated through a supply network ranging from suppliers to end-users [1]. Currently, uncertainty is an inevitable fact in supply chain models. In the aspect of swift technological progressions, the fundamental SCM has tailored rapidly to supply chain networks [2]. A few varying conditions are controllable and assignable while others are uncontrollable and natural. These uncontrollable conditions endanger and challenge the resilience of a supply chain, and can be in the form of environmental and climatic disaster scenarios [3], which negatively affect the global SCM with significant economic losses. For instance, the demand for the traditional drug Radix isatidis encountered disruption during the SARS 2003 epidemic. The belief of the Chinese Government in this drug to combat SARS expanded the demand multi-fold. Likewise, certain production disruptions can be caused by material supply mismatches, labor strikes and machinery breakdowns [4,5]. For instance, back in 2011, tsunami and earthquake disruption instigated Toyota’s auxiliary plants in the areas that produce vehicles and parts to cease operations. In such a situation, an emergency-based SCM with a controllable production rate for flexible manufacturing is an effective solution to deal with maximum disruptions in the chain.

A disaster is either a natural or manmade event that inflicts abrupt and uncontrollable damage throughout a community [6]. Widespread pandemic diseases are perilous forms of disaster, which not only affect a region economically, but importantly lives are lost. Globally, they cause serious macro-economic activity losses during the pandemic and post-pandemic time-frames. Normally, in any disaster scenario, residents expect and require exceptional relief and support, as losses surpass the capability of an affected community to face and meet its demands from normal resources [7]. An outbreak of the recent pandemic; COVID-19 disease and its economic impacts are highly ambiguous, which makes it a daunting task for policymakers to formulate an appropriate macroeconomic response [8]. This perpetrates an immense pressure on policymakers to be preventive and reactive simultaneously at distinct spots. For this purpose, disaster management activities are very essential to assess product needs, forecasting demands, procurement, managing inventory and logistics, and relief distribution to curtail the losses [9].

Previously, researchers i.e., Zavvar et al. [10] have worked on disaster SCM in the pharmaceutical sector by developing a multi-objective model. For Song et al. [11] work is a motivation from real-world disaster scenarios; they developed an SCM model for distribution of medical kits. Cao et al. [12] developed a multi-objective programming model for a relief disbursement supply chain operated over large scale natural disasters. Kaur [13] developed a resilient supply chain model for procurement and logistics during a geothermal disaster. All these studies have been conducted on geothermal disasters, while pandemic is being somehow ignored by researchers at large. During a pandemic, supply chains are surrounded by a number of uncertain variables, which makes it a daunting task to opt for an optimal policy. Thus, understanding uncertainty is vital for controlling policies in an effective SC model. Fuzzy sets-based approaches can be used to overcome these uncertainties in an appropriate manner to ensure practicality in a supply chain.

For uncertain factors during uneven times, flexibility in the operations of a supply chain has developed to be an integral part of research. It can be also utilized to hedge the variability in customer demands in a pandemic situation. For such an objective, productivity levels should be adjusted to match the current demands, in such a way to satisfy the closest customer or allocate priority to higher-margin/critical products [14]. The extent of manufacturing adaptability and flexibility has paved the path to distinct frameworks, and depersonalization like; capacity, production, and logistics flexibility [15]. The novelty about this article is that it targets supply chain management in a pandemic environment (COVID-19) having various scalable emergency levels. Further, non-linear modelling for a controllable production rate is designed embedded with an imperfection model, as proper utilization of the available resources during the pandemic is of prime importance. The costs in the model are set as fuzzy to cope up with the uncertain conditions created by lasting pandemic.

The article is structured in a way i.e., introduction to the supply chain, background to the research article related to SC in pandemics, and its challenges are discussed in this section. In Section 2, the literature is presented through an author contributions table that identifies research gaps. Section 3 presents a detailed analysis of the variable demand, controllable production rate, inventory management, and emergency-based SCM model formulation. Section 4 deals with the numerical experiments, which consist of the required data for performing experiments using the proposed SCM model. The significantly solution methods and numerical results are illustrated in Section 5. Section 6 is utilized to support industrial experts and managers to understand the importance of the study by a proactive analysis during pandemic conditions, whereas the last Section 7 concludes the research study. 

## 2. Related Study

In the past, research studies [7,9,16] analyzed various supply chain models in disaster-based scenarios; while enlightening the traditional deployment of many organizations, they discovered that production policies and inter-organizational integrations are a vital challenge in any disaster management activity. Moreover, in developing regions, several political and socio-economic issues, predominantly, corruption, weak transparency, and non-accountability enormously hinder the triumph of disaster management efforts [17]. A valuable robust supply chain practically emphasizes actual need assessments that fundamentally include a swift, dynamic and coordinated ground evaluation. Though some investigations in developing countries indicate that this assessed demand target been derived from a certain source are randomly reduced forestalling exaggeration of demand; i.e., bullwhip, and no scientific approach is used [18]. Indeed, the robustness of a supply chain is based on the inherent flexibility of the chain [19]. Normal circumstance production is completely different from a disaster-affected one. Hence, for an emergency supply chain management, establishing models, defining roles along with responsibilities, and coordination across every tier of the chain is required, which is an overwhelming task for developing countries [20]. Recently, researchers have identified OR methods for containing the ripple effects generated by the COVID situation [21].

Flexibility in a manufacturing system implies that the production criteria may be altered as desired [22]. A supply chain is flexible in process lead time, production process, production time and rate until it is an in-control state, however, an out-of-control state destabilizes the aforementioned parameters [23]. Flexibility in manufacturing revolves around controllable lead time and cycle time, as it plays a vital role in reducing costs across the supply chain framework. Ouyang et al. [24] studied discrete lead time cash cost in a supply chain management (SCM) system for the first time, where lead time demand was considered as a random variable. Later on, their research was modified in [25], where they injected the concept of reorder point instead of the safety factor. However, both these models assumed setup costs to be constant. Porteus [26] presented the concept of continuous investment for the reduction of setup cost and quality improvement for fundamental economic production quantity (EPQ) model. Taking this concept, Liang-Yuh [27] introduced setup cost reduction and quality improvement in an SCM environment for the first time. Jha and Shanker [28] developed an inventory model with the parameters of controllable lead time and service level constraints. Giri et al. [29] introduced a flexible production rate-based EPQ model, which addressed the issue of higher stress levels of the machinery with the increase in production rate. In this EPQ model, the unit production cost was stated as a function of the production rate, under general failure and overhaul time conditions. Yi and Sarker [30] proposed an SCM model including consignment policy in which a single buyer utilized investments to manipultae lead time, though the production rate was held constant in their study. Li et al. [31] considered lead-time along with risk-averse firm and calculated optimal prices. AlDurgam et al. [32] examined an inventory model considering stochastic demand and controllable production rate along with lead time curtail. Furthermore, Heydari et al. [33] established a coordinate SCM model including lead time reduction cost.

Besides reducing lead times, uncertainty in the demand can be triggered by an uncontrollable factor i.e., in this work the emerging challenge of COVID-19. In such scenarios, controllable production rates are highly effective. Most of the industries across developing countries dependent upon inputs from China, irrespective of their size, started to observe production curtailment. Most significantly, some panic among firms and consumers distorted usual consumption patterns thus creating market anomalies. Only a robust SCM model with flexible and controllable production rates can sustain such a highly unstable market environment. A critical review of a design for robustness in a supply chain network was studied in [34]. Khouja and Mehrez [35] presented the idea of controllable production rates. Christian [36] presented an EPQ model where the production cycle consisted of multiple runs at various production rates. The author revealed that the production rates should take a value between the demand rate and the production rate that reduces the production cost. Later on, Glock (referred to earlier [22]) developed an integrated inventory model with variable production rates. Afterwards, Glock [37] extended his work to a multiple stage system. Moreover, studies of Glock [37,38], also verified that variable production rates either in a single or multi-stage environment imply a significant impact on product defect reduction and system’s cost. Tayyab and Sarkar [39] constructed a multi-stage inventory production model considering the constant rate of production with a random defective rate. Furthermore, Malik and Sarkar [40] developed a model that focused on setup cost reduction, in which controllable lead time with uncertain demand was discussed. There is plenty of work carried on the development of models in SCM with controllable production rates, setup cost reduction, demand constraints, procurement, transportation objective, quality improvement, and lead time crashing costs [40,41,42,43].

During any pandemic, a production environment with defective rates under demand uncertainty is a highly discouraging issue, as resources are of prime importance in an emergency situation. There are various studies about imperfect production, but no link between imperfect production with variable production in a pandemic scenario is found. Khouja and Mehrez (referred to previously [35]) proved the deterioration in the quality of product with an increase in production rates in an economic production inventory model. They reassessed the work of [44,45] and assumed it as a function of quality. Khouja [46] further extended his previous model by assuming that the production rate had a probability to shift production system from in control to the out of control state. Connolly [47] established an automated production setup via integration of the manufacturing system with the SCM framework. The principle benefit of their system was less quantity of defective parts and no requirement for the reduction of setup cost. Later on, Sana [48] studied unit production cost as a function of product reliability and controllable production rate in an imperfect production system. The model was later extended with stochastic demand by [49], sampling in an inspection by [50], and stochastic repair time by [51]. Kim et al. [52] stated that practically shunning defective products must be part of the elementary production system. Sarkar [53] also developed an imperfect production inventory model, assuming a dual-stage assembly operation with backorders and variable production rate. Further, tradeoff between various objectives, and system uncertain analysis in a lifecycle of three-echelon shale gas supply chain has been studied by [54,55,56]. Decision for vendor selection [57], production and distribution [58], pricing strategy [59], and sustainable parameters for stakeholder’s satisfaction [60] are vital studies for providing a baseline of SCM.

Government policies, high absenteeism rate of affected labor, inoperability, and drastic effects on the inflow and outflow of the supply chain may initiate severe economic crises, system failure and even a shutdown. Table 1 summarizes the literature in which various author contributions are presented and it aligns with the claim of the current research. Overall, there has been no research conducted on the integration of an imperfect variable production system operating in a pandemic emergency-based scenario with the supply chain. Considering the abovementioned problems, the current study is concentrated on investigation and inoculation of disaster effected supply chain through flexibility and variability in imperfect production-based sectors of the developing countries. The current study focuses on production rate flexibility to cope up with the uncertainties that emerged from scenarios such as pandemic (e.g., COVID-19) in an economic region.

## 3. Emergency-Based SCM Modelling 

The COVID-19 pandemic created an emergency situation for the supply of raw materials and finished goods, which adversely affected the whole SCM economically and socially. The mathematical model of vendor-manufacturer based SCM is constructed on the management of resources, inventory, analysis of variable demand, controllable production rate, and assumptions, to deal with the distinct levels of the emergency during pandemic. 

### 3.1. Variable Demand Depending Emergency Level

The proposed emergency-based SCM considers variable demand depending on the emergency levels due to the recent COVID pandemic. Initially, it was evident that the outbreak compelled governments to primarily restrict the social contact among people, and gradually closing various activities of public at large, which eventually reached to an extreme emergency level of lockdown. Worldwide, different countries have responded in various manners to the outbreak in terms of the lockdown. Indeed, lockdowns played a beneficial role in controlling the widespread, but as soon as relaxation is provided, high influxes in new cases were observed, for instance in Hong Kong [61]. After the outbreak, China had put sixteen (16) cities in complete lockdown situation, with closure of retailers, transport and other business activities. On the other hand, Sweden, directed its population towards social distancing and preliminary lockdowns [62]. Denmark rushed towards a complete lockdown, and the results appeared fruitful when they removed the restrictions gradually afterwards. Italy and Spain went into preliminary to complete lockdowns, however they suffered a lot from the pandemic spread. The US and UK responded lately with lockdown phases, they sustained a preliminary lockdown for a significant portion of time, however it did not go well for them, and they gradually increased stepwise to full lockdown situations in certain states [63]. 

Various countries adopted different timings and procedures for the lockdown periods, however their levels mostly followed a similar trend. Observing the local, national, and international practices, we came up with setting of different levels after brainstorming with the experts. The emergency levels are different at different locations and cities depending on the policies and guidelines provided by the local government, that is the reason six (6) levels of emergency are developed and linked with the decreasing demand as expressed in the Equations (1) and (2). The six different levels of emergency by controlling COVID-19 are based on real time consequences and are reflecting the movements, transportation, supply, lockdown etc., as given in Table 2.

**Figure 1:**
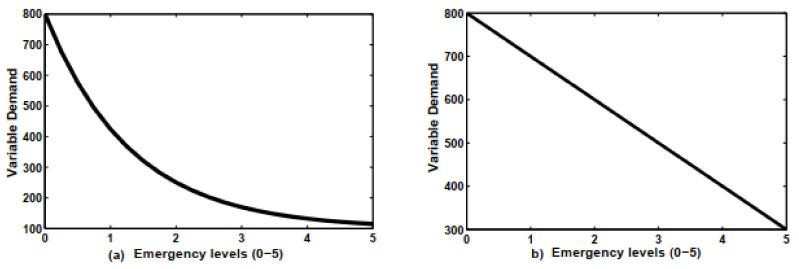
The effect of emergency levels on the market demand: (**a**) is showing an exponentially decreasing demand and (**b**) is representing the linear relationship between emergency levels and market demand.

These actions affect negatively on the market demand of the production and service businesses due to shortages of raw materials caused by supply shortages and customer unavailability in a market. On other hand, certain products in markets were saturated due to unavailability of customers to buy (decreasing demand) due to the pandemic, which created an economic anomaly for businesses and governments. Therefore, to justify the decreasing demand as a function of the emergency levels, decreasing linear and exponential equations are generated. The idea of decreasing linear and exponential functions is taken from the research work done by [64,65,66]. The linear equation is as follows:

(1) $$D(\Omega )=\sigma_{1}-\rho_{1}\Omega$$

where, D(Ω) is the variable demand depending emergency level (Ω) due to the pandemic, $\rho_{1}$ is the lowest possible market demand and scaling factor, $\sigma_{1}$ is the initial demand. The market demand can be affected exponentially by the increasing level of the emergency due to pandemic situation. The representation is well given by the equation given as follows:

(2) $$D(\Omega )=\rho_{2}+\sigma_{2}e^{-\Omega \lambda }$$

where, $\rho_{2}$ is the lowest possible market demand, $\sigma_{2}$ is the scaling factor, and λ is the shape factor. The graphical representations of the exponentially and linearly decreasing demand by the emergency levels due to pandemic are shown in Figure 1.

### 3.2. Fuzzy Costs Theory for Uncertain Environment

The COVID-19 pandemic created an uncertain environment for supply and demand of goods, which is dangerous for global SCM. Numerous uncertainties are possible in real supply chain management problems, but they are modeled traditionally using approaches derived from probability theory. However, there are undeniable uncertainties, which cannot be controlled optimally using probabilistic models. This issue arises in model problems in inventory, production, and SCM under uncertain environments to find optimal solutions [67]. Therefore, the solution to deal such problems is with fuzzy set theorems, rather than probability theory [68]. Vujosevic et al. [69] developed the inventory models in a fuzzy sense where ordering cost was represented as a triangular fuzzy and holding cost by a trapezoidal fuzzy number. Therefore, an SCM model with emergency conditions due to COVID-19 pandemic considering basic costs in fuzzy logic where the signed distance method is used for defuzzification. 

In order to fuzzify all the basic inventory and production costs involved in the emergency based SCM, we consider triangular fuzzy numbers. The distance of triangular fuzzy number x is given in Equation (4) as follows:

(3) $$d(\tilde{x},{\tilde{0}}_{1})=x+\frac{1}{4}(\Delta_{2}-\Delta_{1})$$

where x be a triangular fuzzy number, $\tilde{x}=(x-\Delta_{1},x,x+\Delta_{2}$) where $0<\Delta_{1}<x$ and $0<\Delta_{2}\leq 1-x$. Further, $\Delta_{1}$ and $\Delta_{2}$ are determined by the decision makers.

### 3.3. Supply Chain between Vendor and Manufacturer

The supply chain management model is based on a multi-stage imperfect manufacturer and vendor with controllable production rates, the flow diagram of the firms manufacturing process. The first stage includes the basic manufacturing operations, the second stage is dedicated to the vendor’s manufacturing, and the third stage is the finishing stage of the manufacturer. Due to the capacity constraints of the manufacturing firm, few processing steps of each product are delivered to the vendor for manufacturing. The raw materials in a manufacturing firm are first processed through the basic manufacturing operations, then the semi-finished parts are transported to the vendor. The inspection operations are carried out, where the parts are sorted into good and defective parts. The defective rate is considered a function of occupational stress among workers’ and is directly proportional to it. The defective parts are returned for reworking operations, and again inspected afterwards to sort out the rejected parts, which are eliminated from the flow of production or disposed of in some way. In order to compensate the rejection to meet the required demand, the same quantity of rejections is ordered to be manufactured from first stage of the supply chain management chain. The good parts are further delivered to the final stage of the supply chain management chain for further processing.

### 3.4. Inventory Management of the Vendor-Manufacturer SCM

Vendor-manufacturer-based supply chain management deals with the complex operations by the combination of labor, machine cells and materials. It includes the complex management of inventory between vendor and manufacturer. The inventory diagram of the supply chain management considerers an imperfect production system, where the SCM cycle time is given on the x-axis and inventory is given on the y-axis. The upper portion shows the inventory of the manufacturing firm while the lower portion consisting of reworking operations along with buffer storage is associated with the vendor inventory.

The inventory of the supply chain management is based on multi-stage manufacturing, where the manufacturer’s first stage production is done with respect to *P_ja_* production in cycle time *t*_1_*_j_* and *I_max_*_(*ja*)_ is the maximum inventory. Afterward, parts are transported to the vendor for the remaining operations of the outsourcing process. Here, the parts are inspected and sorted in parallel to the production rate of *P_jb_*(1 − *α_j_*) in *t*_2_*_j_*. The reworking parts *α_j_Q_j_* are inspected and are recycled with the production rate of *P_jb_*(1 − *α_j_β_j_*) in cycle time *t*_3*j*_ where the maximum inventory is denoted by *I_max_*_(*jb*)_. The rejection rate after inspection is formulated as *α_j_β_j_Q_j_*. In this direction, the inventory is transferred again to the manufacturing site for the final and finishing stage of the manufacturer by attaining the production rate of *P_jc_* − *D_j_* to reach to the maximum inventory of *I_max_*_(*jc*)_ in cycle time *t*_4_*_j_* + *t*_5_*_j_*. The equations for each of cycle time are given as:

$$\begin{array}{l} t_{1j}=\frac{Q_{j}}{P_{ja}},\\ t_{2j}=\frac{Q_{j}}{P_{jb}},\\ t_{3j}=\frac{Q_{j}\alpha_{j}}{P_{jb}},\\ t_{4j}=\frac{Q_{j}}{P_{c}},\\ t_{5j}=\frac{Q_{j}}{D_{j}(\Omega )}\left(1-\frac{D_{j}(\Omega )}{P_{jc}}\right). \end{array}$$

### 3.5. Assumptions of the Model

The following assumptions were used for the proposed model.

(1) The SCM mathematical model is based on multiple types of products. [I] The controllable production rate is considered to cope with varying demand. [II] The demand is variable and depending on the emergency level due to pandemic based disaster. [III] The unit production rate of the system is taken as variable, which is depending on the production cost of the manufacturing firm.
(2) The manufacturer deliver product to the vendor due to limited constraint. The imperfect products are produced, for which reworking is done and inspection cost is incurred. The rejected products are recycled and disposed.
(3) The production model is carried out for parallel machining process.

### 3.6. Notation

The decision variables and the parameters used in the proposed mathematical modelling of the SCM are denoted by the notations listed as follows:
*Indices**j*:The index used to indicate number of products, *j* = 1, 2, ... *N**a*:To indicate the parameters for first stage of manufacture *b*:Used with the vendor parameters *c*:To represent the final stage of manufacture*Decision variables**Tj*: Cycle time to manufacture the *j-*th product (units)*Lja*:Labors utilized at 1st stage to manufacture the *j-*th product (workers)*Ljc*:Labors utilized to manufacture the *j*-th product at 1st during final stage (workers)*Kja*:Number of machine units utilized at the 1st stage to manufacture the *j-*th product (workstations)*Kjc*:Number of machine units utilized to manufacture the *j-*th product during the finishing stage (workstations)*Pja*:Plant production rate of the *j*-th product in the 1st stage (units/year)*Pjc*:Plant production rate of the *j-*th product in the 2nd stage of manufacture (units/year)*Manufacturer Parameters**Crm*Raw material cost of the *j-*th product ($/unit)*TDmj*Manufacturer total tool-die cost of the *j*-th product ($/unit)*TDmaj*Tool-die cost of the *j*-th product in 1st stage of manufacture ($/unit)*TDmcj*Tool-die cost of the the *j-*th product in final stage ($/unit)*gj*Total indirect production cost of the *j*-th product ($/unit)*gmaj*Indirect cost of the *j-*th product in first stage of manufacture ($/unit)*gmcj*Indirect production cost of *j*-th product in the final stage of manufacture ($/unit)*Dj*(Ω)Variable demand depending emergency level due to pandemic ($/unit)*Qj*Production quantity (units)*lja*Average labor utilized per machine in first stage of manufacture (labor/machine)*ljc*Average labor utilized per machine in final stage of manufacture (labor/machine)*Wj*Average wedge of labor to manufacture the *j*-th product ($/labor)*Aj*Setup cost for *j*-th product ($/year)*hmj*Manufacturer’s holding cost of each product per cycle ($/unit/year)*Gj*Reworking cost of the *j*-th product ($/unit)*εja*Production rate of each machine unit to manufacture the *j*-th product in the 1st stage (units/machine)*εjc*Production rate of each machine unit at final stage to manufacture the *j-*th product (units/machine)*TCmj*Total cost of manufacture ($/cycle)*Vendor Parameters**TD_bj_*Vendor tool-die cost of the *j*-th product ($/unit)*G_bj_*Indirect part production cost of the *j*-th product with the vendor ($/unit)*θ_j_*Fixed inspection cost of the *j*-th product ($/year)*α_j_*Proportion of scrap produced in a defective *j*-th product (%)*β_j_*Defective rate for the *j*-th product (%)*ψ_j_*Variable inspection cost of the *j*-th product ($/unit)*R_j_*Reworking cost of the *j*-th product ($/unit)*γ*_1_Scrap disposal cost ($/unit)*ρ*Efficiency of the labor (%)*P_jb_*Production rate of jth product manufactured by the vendor (units/year)*TC_bj_*Total cost of the vendor ($/cycle) MR marginal rate of the vendor ($/cycle) *Other Parameters*∆_2_The maximum possible value of the parameters ($)∆_1_The minimum possible value of the parameters ($)*TCj*Total cost of supply chain management for the *j*-th product ($/cycle)*TI*Total inventory*A*Area

### 3.7. Mathematical Model Formulation

The basic costs related to inventory and production are uncertain because of pandemic situation created as a result of COVID-19. Not considering these uncertain conditions results in an unreliable supply chain (SC) model. For this reason, all basic costs associated with the vendor and manufacturer the proposed model are considered as fuzzy costs. The signed distance formula is utilized to solve the fuzzy sets of parameters. The total cost of SCM is the sum of manufacturer cost and vendor cost as given in Equation (4) and their formulations are further represented as follows:

*TC_j_* = *TC_mj_* + *TC_vj_* (4)

#### 3.7.1. Manufacturer’s Cost

The total cost associated with the manufacturer include the cost related to the first stage and final stage of the SCM, where the basic costs are setup, production, labor, holding, and carbon emission as expressed in Equation (5):

(5) $$\begin{array}{c} \mathrm{Total}\;\mathrm{cost}\;\mathrm{of}\;\mathrm{manufacturer}=\mathrm{Seup}\;\mathrm{cost}+\mathrm{Manufacturing}\;\mathrm{cost}+\mathrm{Labor}\;\mathrm{cost}+\mathrm{Holding}\;\mathrm{cost}\\ +\;\mathrm{Carbon}\;\mathrm{emissions}\;\mathrm{cost} \end{array}$$

The breakup of the manufacturer’s total costs is necessary to understand each cost clearly. That is the reason, all the costs are described mathematically and theoretically.

##### Setup Cost

(6) $$A_{j(fuzzy)}=\sum_{j=1}^{J}(A_{j}+\frac{1}{4}(\Delta_{aj2}-\Delta_{aj1}))$$

##### Controllable Production Rate

Cycle time is taken as a decision variable in the SCM model, which is dependent upon the production rates of the manufacturing system. The plant production rates of the manufacturer, i.e., *P_ja_* and *P_jc_* for the first and final stage, rely on the production rate of the machines ($\varepsilon_{ja}^{*}$ and $\varepsilon_{jc}^{*}$). In order to meet the decreasing demand as a variable depending emergency level in disaster or pandemic situation, the production rates are considered as a variable (i.e., $\varepsilon_{ja}^{*}$ and $\varepsilon_{jc}^{*}$) to take an advantage of flexible production, where, $\varepsilon_{ja}^{*}\in [\varepsilon_{ja-min}^{*},\varepsilon_{ja-max}^{*}]$ and $\varepsilon_{jc}^{*}\in [\varepsilon_{jc-min}^{*},\varepsilon_{jc-max}^{*}]$. The production rates of the manufacturer in the first and final stage can be expressed as $P_{ja}^{*}=K_{ja}^{*}\varepsilon_{ja}^{*},$ and $P_{jc}^{*}=K_{jc}^{*}\varepsilon_{jc}^{*}.$ where, $K_{ja}^{*}$ and $K_{jc}^{*}$ are the number of optimal machines required in the first stage and final stage of the manufacturer in SCM.

##### Production Cost

The cost of production is the sum of all the costs associated with the raw material, indirect cost, and tool-die cost [51]. The expression of fuzzy production cost is given in Equation (6a).

##### Production Cost

(6a) $$\begin{array}{c} =\sum_{j=1}^{J}[(C_{rm}+\frac{1}{4}(\Delta_{crm2}-\Delta_{crm1}))+(TD_{maj}+\frac{1}{4}(\Delta_{tma2}-\Delta_{tma1}))P_{ja}+\frac{\left(g_{maj}+\frac{1}{4}(\Delta_{ga2}-\Delta_{ga1})\right)}{P_{ja}}]\\ .P_{ja}t_{1j}+[(TD_{mcj}+\frac{1}{4}(\Delta_{tmc2}-\Delta_{tmc1}))P_{jc}+\frac{\left(g_{mcj}+\frac{1}{4}(\Delta_{gc2}-\Delta_{gc1})\right)}{P_{jc}}]Q_{j} \end{array}$$

##### Holding Cost

The holding cost will be applied on the inventory supported by manufacturer and vendor. The average inventory is calculated as the ratio of the sum of inventories in the form of area under the curve to the cycle time of the production. The cycle time and inventory levels of the production system are calculated by step by step procedure. The holding cost is expressed as follows:

*TI* = *A*_123_ + *A*_10,11,12_ + *A*_11,12,13_ (6b)

(6c) $$\begin{array}{c} =\frac{Q_{j}^{2}}{2P_{ja}}+\frac{{\left(Q_{j}-u\right)}^{2}}{2P_{jc}}(1-\frac{D_{j}}{P_{jc}})+\frac{{\left(Q_{j}+u\right)}^{2}}{2D_{j}}{\left(1-\frac{D_{j}}{P_{jc}}\right)}^{2}\\ \mathrm{Holding}\;\mathrm{cost}=\sum_{j=1}^{J}(h_{mj}+\frac{1}{4}(\Delta_{hm2}-\Delta_{hm1}))[\frac{Q_{j}^{2}}{2P_{ja}}+\frac{{\left(Q_{j}-u\right)}^{2}}{2P_{jc}}(1-\frac{D_{j}}{P_{jc}})+\frac{{\left(Q_{j}+u\right)}^{2}}{2D_{j}}{\left(1-\frac{D_{j}}{P_{jc}}\right)}^{2}]. \end{array}$$

##### Carbon Emission Costs

Carbon emissions are generated during manufacturing of products in the production system, which significantly affect people, society and the environment. The cost of carbon is incurred by state governments to address the global warming issue. The proposed model considers the carbon emission cost, which is the function of the production rate of the system. The cost of carbon emissions is generated during the life cycle production where *A* is the emissions function parameter (ton·year/unit^3^), *B* is the emissions function parameter (ton⋅year/unit^2^), and *C* is the emissions function parameter (ton/unit) [62]. The corresponding Equation (6d) is as follows:

(6d) $$\mathrm{Carbon}\;\mathrm{Emission}\;\mathrm{Cost}\;=\sum_{j=1}^{J}\gamma_{2}[(AP_{ja}^{2}-BP_{ja}+C)P_{ja}t_{1j}+(AP_{jc}^{2}-BP_{jc}+C)Q_{j}].$$

##### Labor Cost

This cost is associated with the utilization of the workforce in the production system. The wages are paid to the workers on the basis of their skill levels. Here, the cost is incurred to reflect the importance of the unskilled workers to understand the importance of the human factor in the production system. The labor cost is calculated on the basis of the machines required in the stages of the supply chain management, which is expressed in fuzzy form as in Equation (7):

(7) $$\mathrm{LC}=\sum_{j=1}^{J}L_{j}(W_{j}+\frac{1}{4}(\Delta_{w2}-\Delta_{w1}))$$

The number of machines and the number of laborers required for the production system are expressed below as:

(7a) $$K_{j}=K_{ja}+K_{jc}$$

(7b) $$L_{j}=L_{ja}+L_{jc},$$

(7c) $$\begin{array}{c} \mathrm{Number}\;\mathrm{of}\;\mathrm{labors}\;=\;\mathrm{Labor}\;\mathrm{rate}\;\times \mathrm{Number}\;\mathrm{of}\;\mathrm{machines}\;÷\mathrm{labor}\;\mathrm{efficiency}\\ L_{j}=\frac{l_{ja}}{\rho }K_{ja}+\frac{l_{jc}}{\rho }K_{jc} \end{array}$$

where, *Lj* is the number of labors and *Kj* is the number of machines and where, *I_ja_* and *I_jc_* are the labor rate or the number of laborers working on each machine while *ρ* is the efficiency of the workers. Therefore, the mathematical form of the total cost of manufacturer in the fuzzy system is expressed as in Equation (8). The tilde sign is used for parameters considering fuzzy (numbers) costs:

(8) $$\begin{array}{c} \tilde{T}C_{m}=\sum_{j=1}^{J}[(A_{j}+\frac{1}{4}(\Delta_{aj2}-\Delta_{aj1}))+((C_{rm}+\frac{1}{4}(\Delta_{crm2}-\Delta_{crm1}))+(TD_{maj}+\frac{1}{4}(\Delta_{tma2}-\Delta_{tma1}))P_{ja}\\ +\frac{\left(g_{maj}+\frac{1}{4}(\Delta_{ga2}-\Delta_{ga1})\right)}{P_{ja}})P_{ja}t_{1j}+((TD_{mcj}+\frac{1}{4}(\Delta_{tmc2}-\Delta_{tmc1}))P_{jc}+\frac{\left(g_{mcj}+\frac{1}{4}(\Delta_{gc2}-\Delta_{gc1})\right)}{P_{jc}})\\ Q_{j}+L_{j}(W_{j}+\frac{1}{4}(\Delta_{w2}-\Delta_{w1}))+(h_{mj}+\frac{1}{4}(\Delta_{hm2}-\Delta_{hm1}))[\frac{Q_{j}^{2}}{2P_{ja}}+\frac{{\left(Q_{j}-u\right)}^{2}}{2P_{jc}}(1-\frac{D_{j}}{P_{jc}})+\frac{{\left(Q_{j}+u\right)}^{2}}{2D_{j}}\\ {\left(1-\frac{D_{j}}{P_{jc}}\right)}^{2}]+\gamma_{2}[(AP_{ja}^{2}-BP_{ja}+C)P_{ja}t_{1j}+(AP_{jc}^{2}-BP_{jc}+C)Q_{j}]]. \end{array}$$

#### 3.7.2. Vendor Cost

The semi-finished products are delivered to the vendor to perform operations. The costs of the vendor are the sum of the costs associated with the production, holding, inspection, rework, and disposal, given in Equation (9):

(9) Vendor cost=Production cost+Holding cost+Inspection cost+Reworking cost+Scrap disposal cost+Buffer cost.

##### Production Cost of Vendor

The expression for production cost utilizes the value from the research work done by [53], except for the cost of raw materials, because the semi-finished products are received from the manufacturer. The fuzzy cost expression is as given in Equation (10):

(10) $$=\sum_{j=1}^{J}[[(TD_{oj}+\frac{1}{4}(\Delta_{to2}-\Delta_{to1}))P_{jb}(1-\alpha_{j})+\frac{\left(g_{oj}+\frac{1}{4}(\Delta_{go2}-\Delta_{go1})\right)}{P_{jb}(1-\alpha_{j})}]P_{jb}(1-\alpha_{j})t_{2j}].$$

##### Holding Cost of Vendor

The holding cost of the vendor is obtained from the sum of inventories. The corresponding fuzzy expression is represented by Equation (11):

(11) $$\begin{array}{c} \mathrm{Total}\;\mathrm{Inventory}=\;{\mathrm{Area}}_{456}\;+\;{\mathrm{Area}}_{5678}\;+\;{\mathrm{Area}}_{689}\;\\ =\frac{Q_{j}^{2}(1-\alpha_{j})}{2P_{jb}}+\frac{\alpha_{j}(1-\alpha_{j})Q_{j}^{2}}{P_{jb}}+\frac{\alpha_{j}^{2}Q_{j}^{2}(1-\alpha_{j}\beta_{j})}{2P_{jb}},\\ \mathrm{HC}=\sum_{j=1}^{J}[(h_{bj}+\frac{1}{4}(\Delta_{hb2}-\Delta_{hb1}))[\frac{Q_{j}^{2}(1-\alpha_{j})}{2P_{jb}}+\frac{\alpha_{j}(1-\alpha_{j})Q_{j}^{2}}{P_{jb}}+\frac{\alpha_{j}^{2}Q_{j}^{2}(1-\alpha_{j}\beta_{j})}{2P_{jb}}]]. \end{array}$$

##### Inspection Cost

Inspections are carried out, where the products are checked according to the established quality control dimensions and checks. The parts are categorized into good, rejected and rework parts. Total inspection cost (*IC_j_*) of the production is the sum of the fixed and variable inspection cost in the production, expressed by Equation (12):

(12) $$=\sum_{j=1}^{J}[\theta_{j}+\psi_{j}P_{ja}t_{1j}+\psi_{j}P_{jb}t_{2j}].$$

##### Reworking Cost

Inspections are carried out to inspect defective products along the production flow. Rework parts are returned to the same workstation for processing, which involves costs of processing, energy, labor, etc.:

(13) $$\begin{array}{c} =\sum_{j=1}^{J}R_{j}I_{maxjba},\\ =\sum_{j=1}^{J}R_{j}\alpha_{j}Q_{j}(1-\alpha_{j}\beta_{j}). \end{array}$$

##### Disposal Cost

Rejected products or scraps are obtained after inspection operations and are discarded. As a result, a cost is incurred due to the applies disposal process or recycling given as:

(14) $$=\sum_{j=1}^{J}\gamma_{1}P_{jb}t_{2j}\alpha_{j}\beta_{j}.$$

The mathematical expression for fuzzy vendor cost to sum all the costs equations is represented as given in Equation (15):

(15) $$\begin{array}{c} \tilde{T}C_{b}=\sum_{j=1}^{J}[((TD_{bj}+\frac{1}{4}(\Delta_{tb2}-\Delta_{tb1}))P_{jb}(1-\alpha_{j})+\frac{\left(g_{bj}+\frac{1}{4}(\Delta_{gb2}-\Delta_{gb1})\right)}{P_{jb}(1-\alpha_{j})})P_{jb}(1-\alpha_{j})t_{2j}\\ +(h_{oj}+\frac{1}{4}(\Delta_{hb2}-\Delta_{hb1}))(\frac{Q_{j}^{2}(1-\alpha_{j})}{2P_{jb}}+\frac{\alpha_{j}(1-\alpha_{j})Q_{j}^{2}}{P_{jb}}+\frac{\alpha_{j}^{2}Q_{j}^{2}(1-\alpha_{j}\beta_{j})}{2P_{jb}})\\ +\theta_{j}+\psi_{j}P_{ja}t_{1j}+\psi_{j}P_{jb}t_{2j}+R_{j}\alpha_{j}Q_{j}(1-\alpha_{j}\beta_{j})+\gamma_{1}P_{jb}t_{2j}\alpha_{j}\beta_{j}] \end{array}$$

The production system of the manufacturing firm is analyzed by the formulation of the mathematical model, which is based on the cycle time of production. The objective of the proposed model is to minimize the total cost of SCM, which is given as the following fuzzy expression as:

$$\tilde{T}C_{j}=\tilde{T}C_{m}+MR\times (\tilde{T}C_{v})$$

where, MR is the marginal rate of the vendor. Hence the mathematical expression of the objective function is given by Equation (11):

(16) $$\begin{array}{c} TC_{j}=\sum_{j=1}^{J}\frac{1}{T_{j}}[(A_{j}+\frac{1}{4}(\Delta_{aj2}-\Delta_{aj1}))+((C_{rm}+\frac{1}{4}(\Delta_{crm2}-\Delta_{crm1}))+(TD_{maj}+\frac{1}{4}(\Delta_{tma2}-\Delta_{tma1}))P_{ja}\\ +\frac{\left(g_{maj}+\frac{1}{4}(\Delta_{ga2}-\Delta_{ga1})\right)}{P_{ja}})P_{ja}t_{1j}+((TD_{mcj}+\frac{1}{4}(\Delta_{tmc2}-\Delta_{tmc1}))P_{jc}+\frac{\left(g_{mcj}+\frac{1}{4}(\Delta_{gc2}-\Delta_{gc1})\right)}{P_{jc}})Q_{j}\\ +L_{j}(W_{j}+\frac{1}{4}(\Delta_{w2}-\Delta_{w1}))+(h_{mj}+\frac{1}{4}(\Delta_{hm2}-\Delta_{hm1}))[\frac{Q_{j}^{2}}{2P_{ja}}+\frac{{\left(D_{j}T_{j}\right)}^{2}}{2P_{jc}}(1-\frac{D_{j}}{P_{jc}})+\frac{{\left(Q_{j}+u\right)}^{2}}{2D_{j}}{\left(1-\frac{D_{j}}{P_{jc}}\right)}^{2}]\\ +\gamma_{2}P_{ja}t_{1j}[(AP_{ja}^{2}-BP_{ja}+C)+(AP_{jc}^{2}-BP_{jc}+C)Q_{j}]+s.SC_{j}+MR[((TD_{bj}+\frac{1}{4}(\Delta_{tb2}-\Delta_{tb1}))(1-\alpha_{j})\\ P_{jb}+\frac{\left(g_{bj}+\frac{1}{4}(\Delta_{gb2}-\Delta_{gb1})\right)}{P_{jb}(1-\alpha_{j})})P_{jb}(1-\alpha_{j})t_{2j}+(h_{bj}+\frac{1}{4}(\Delta_{hb2}-\Delta_{hb1}))[\frac{Q_{j}^{2}(1-\alpha_{j})}{2P_{jb}}+\frac{\alpha_{j}(1-\alpha_{j})Q_{j}^{2}}{P_{jb}}\\ +\frac{\alpha_{j}^{2}Q_{j}^{2}(1-\alpha_{j}\beta_{j})}{2P_{jb}}]+\theta_{j}+\psi_{j}P_{ja}t_{1j}+\psi_{j}P_{jb}t_{2j}+R_{j}\alpha_{j}Q_{j}(1-\alpha_{j}\beta_{j})+\gamma_{1}P_{jb}t_{2j}\alpha_{j}\beta_{j}]] \end{array}$$

where:

$$L_{j}=L_{ja}+L_{jc}$$

$$Q_{j}=\frac{T_{j}D_{j}(\Omega )}{1-\alpha_{j}^{2}\beta_{j}}$$

$$t_{1j}=\frac{T_{j}D_{j}}{K_{ja}\varepsilon_{ja}(1-\alpha_{j}^{2}\beta_{j})}$$

$$t_{2j}=\frac{T_{j}D_{j}}{P_{jb}(1-\alpha_{j}^{2}\beta_{j})}$$

$$t_{4j}=\frac{T_{j}D_{j}}{K_{jc}\varepsilon_{jc}}$$

$$t_{5j}=(\frac{T_{j}D_{j}}{1-\alpha_{j}^{2}\beta_{j}}-u)(\frac{K_{jc}\varepsilon_{jc}-D_{j}}{D_{j}K_{jc}\varepsilon_{jc}})$$

The SCM mathematical model is non-linear by minimizing total cost of SCM, where the decision variables are $(T_{j}$, $L_{ja}$, $L_{jc}$, $K_{ja}$, $K_{jc}$, $P_{ja}$, and $P_{jc})$.

## 4. Numerical Experiments

The model is formulated for emergency-based SCM consisting of suppliers and manufacturers. The variabilities in the proposed model make it non-linear in nature, where the production function constraint is non-linear. The decision variables considered rely on the implemented production planning decisions. The pragmatic application of the proposed emergency-based SCM is proposed to face variable demand situations. To avoid shortages, the managers are required to keep the production rate as a controllable feature to be set as per demand. On the other hand, the demand is also variable and dependent on the level of emergency incurred due to the pandemic conditions in the disaster. The production rate of the manufacturing system is linked with the integrated production rate of man and machine. Therefore, it is limited to setting the production rate of the operations before delivering the products to the vendor which should be greater than the vendor’s production rate, and furthermore, the rate of production operations performed during the 2nd stage of the manufacturer must be greater than the predecessor. The variable production system is considered for multi-product and multi-stage production to minimize the total cost of SCM.

To set the model application in real life scenario, an automobile part manufacturing industry is considered. The production of the industry is purely discrete; manufacturing three parts i.e., *A*, *B* and *C*. The data utilized to perform the experiment is taken from the research described in [70,71,72]. The manufacturing-based data for each product, given in Table 3, consist of tool-die, production, holding and production rate, which is taken from the research work of [53]. On the basis of the capacity of the machines inside manufacturing firm, the controllable production rate of the machines at first stage i.e., $\left[\varepsilon_{ja-min},\varepsilon_{ja-max}\right]$ are considered as (120, 150), (130, 160), (140, 170) and final stage i.e., $\left[\varepsilon_{jc-min},\varepsilon_{jc-max}\right]$ are considered as (110, 130), (115, 140), and (125, 150) to manufacture parts *A*, *B* and *C*, respectively.

All the data related to the imperfect item production are given in Table 4, which covers the inspection, reworking, recycling and buffer costs. The reworked parts again bear an extra cost in the form of energy, labor and machine costs called reworking cost. The inspection cost is categorized as fixed, including the initial investment and variable cost depending upon the production quantity. The recycling cost includes the operations to dispose the given rejected products which cannot be reworked in some way. These costs having a significant impact on the total cost of production.

## 5. Numerical Results

The objective is to make the production flat, where the number of workstations, workers, and production time cycle are required. The production rate of the system depends upon the production rate of the machines, which is kept in such a way that there are no shortages in the system. The systems of equations generated from the proposed model consist of non-linear equations, which are complex enough to solve using analytical methods. There are numerous techniques that can be used to find the optimal solution of non-linear models, e.g., interior point optimization (IPO), particle swarm optimization (PSO), pattern search (PS), genetic algorithm (GA), min-max optimization (MMO), etc. All these algorithms are available in the OPTIMTOOL application of the MATLAB software package, but it requires an MATLAB code to be generated in the M-file of the package.

Analytically, the methodology is applied to search the optimal and global solutions. However, these analytical methods are time consuming and ineffective, and have been largely replaced by techniques based on quadratic programming. These methods as sequential quadratic programming (SQP), which are based on Newton’s method to deal with the unconstrained optimizations and identified as a most effective method to solve big-data problems. Schittkowski [73] validated these methods on the basis of successful solutions and proved they have good accuracy for big-data research problems [74]. The works of Biggs [75,76] ensure that the SQP method implements the Newton’s method for constrained optimization in an effective way when solving unconstrained optimization problems [77]. The proposed model is coded in MATLAB 2017b to find the optimal result and the solution using SQP as given in Table 5. A sequential quadratic programming (SQP) method is utilized, which is available in the OPTIMTOOL of the MATLAB 2017b version. Firsts of all, the two type of demands i.e., exponential and linear are considered for the calculation of the minimum total cost (*TC_j_*) of SCM:

(1) It is found that in case of exponentially decreasing demand, the optimal *TC_j_* of SCM is obtained as $124835.5, where the cycle time are (13.68, 15.264, 15) days cycle, optimal machine utilization are (4, 3, 3) for first stage and (4, 4, 4) for final stage, labor required are (10, 7, 7) for first stage and (10, 10, 10) for final stage, and optimal production rate to be set are (600, 480, 510) and (520, 560, 600) units/cycle for manufacturing parts *A*, *B* and *C,* respectively.
(2) On the other hand, the optimal *TC_j_* with linear demand is calculated as $226282.3. The results are well evaluated against solution methodology of PS and GA as an evidence. The possible optimal production plan for manufacturing of parts A, B, and C as a solution consider production cycle time (7.92, 8, 8) days, machines utilization (6, 5, 5) at first stage and (7, 6, 6) at final stage of manufacturing respectively. The indirect decision variable i.e., labor required, are calculated as (15, 12, 12) and (17, 15, 15) whereas optimal controllable production rate is required to set at (900, 800, 850) and (910, 840, 900) for first and final stage of manufacture, respectively.

## 6. Managerial Insights

The COVID-19 pandemic created a huge disruption affecting governments, industries, supply chains, businesses, retailers and people economically. This negative impact continues to increase until it is controlled. The reason is that the product demand is highly uncertain and is dependent on the actions of the local government, i.e., to control the transmission of the virus among people. In this hard time, the industrial managers and experts should make proactive decisions to avoid huge SCM economic losses. These decisions include variable production rates, machine utilization, required labor force, and inventory levels. Therefore, the proposed emergency-based SCM model provides a platform for managers regarding these important decisions during different conditions implemented by the government due to the ongoing pandemic. These optimal results and optimal solutions are presented in Table 6. The demand considered in this analysis is decreasing linearly against emergency levels from 0 to 5.

**Figure 2:**
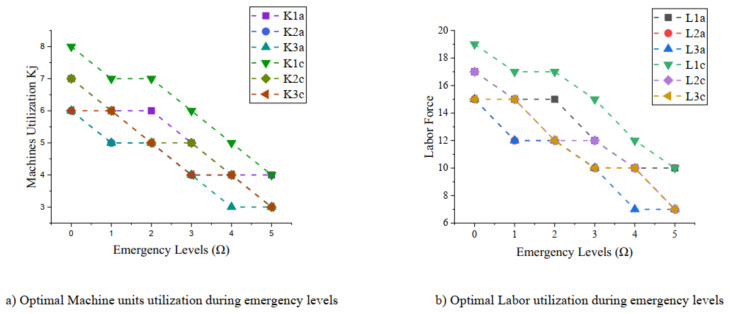
The optimal utilization of the resources in pandemic conditions: (**a**) Machines utilization (**b**) Labor force required.

**Figure 3:**
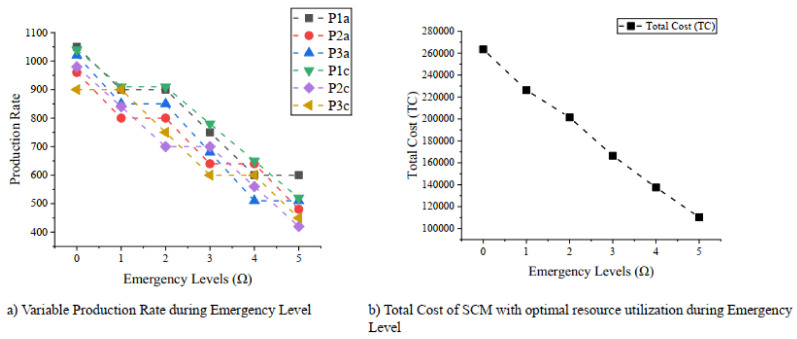
The representation of plant production rate and total cost during emergency: (**a**) is showing the optimal controllable production rate required by manufacturing plant (**b**) is the representation of decreasing total cost of SCM by the proactive approach to cope with variable demand.

These results are discussed well for understanding them by the following managerial insights:

(1) The first analysis is about the optimal utilization of resources, i.e., machine units and the required labor force. The analysis is represented graphically in Figure 2. The left-hand side curve in the figure shows the relationship between machine units (K_j_) and emergency levels (Ω), where the manufacturer is required to utilize optimal machines for manufacturing products *A*, *B*, and *C* in each stage, respectively, to cope with decreasing dependent demand. The analysis of the required labor force (L_j_) is illustrated on the right-hand side of the Figure 2, where the relationship is again inversted to justify the market demand from emergency level 0 to 5. From this analysis, it is important for managers to avoid extra production and excess supply by the optimal utilization of workers and machines during various emergency situations due to COVID-19.
(2) The second insight reflects the significance of controllable production rates. The proposed research model is solved by considering controllable production rates of the manufacturing plant during the first stage and final stage of the SCM. The value of controllable production rate is the input given by the production manager after analyzing the market demand. During COVID-19, this is a challenging task for decision makers to set and control production rate with respect to the extreme variation in the market demand. The analysis of the controllable production rate (P_j_) for first and final stage of manufacturer is well illustrated against increasing emergency level (Ω) on the left-hand side of the Figure 3. The production rate should be set decreasing with respect to the decreasing market demand to avoid cost of production. Similarly, the analysis of the total cost of SCM (TC_j_) is represented at right hand side of the Figure 3. It is found that the total cost of production can be optimized at minimum level during emergency level by controlling production rate and production resources.

## 7. Conclusions

The resilience of supply chain management (SCM) is badly affected by the current novel COVID-19 pandemic, which causes emergencies due to the varying demand and supply shortages. Various emergencies resulting due to pandemic are examined and analyzed. An emergency-based SCM is developed for the uncertain product supply and inventory management situation between vendors and manufacturers with imperfections. This research aims to help decision-makers and managers cope with the consequences and global disruption created by the COVID-19 pandemic. A pragmatic application of the model is justified by undertaking an automobile part manufacturing industry as a exemplary manufacturer in SCM for flexible production as abrupt demand surges in the manufacturing sector have been indicated by recent studies [78] and in particular in the automobile sector [79]. A timely need to mitigate costly effects was required. Further, the demand is variable and uncertain during different emergency situations of the COVID resulting from government actions. For this purpose, an optimal total cost of SCM is obtained by solving a non-linear mathematical model using SQP methodology with linear and exponential demand. As the pandemic is of a highly uncertain nature, fuzziness is incorporated to deal with the possible fluctuations in costs and positive and negative demand surges. The results reflect the best utilization of the machine units, labor force, and controllable production rate against various emergency levels for flexible manufacturing. A set of sensitivity experiments allows us to illustrate the behavior of the proposed SCM model and to derive useful insights. More specifically, the study is a proactive approach for decision-makers to take advantage of the controllable production rate to avoid excess supply against decreasing demand with the minimum optimal cost of SCM.

The solution of the research is provided by incorporating a controllable production rate for flexible manufacturing, inventory level control, and best resource utilization to cope with the fluctuating demand due to the emergency levels determined by the government to control the transmission of the virus. The results of the research show a deep knowledge of the varying demand concerning the emergency level in a pandemic. The research is effective for governments and disaster management stakeholders to understand the consequences of emergency levels during a pandemic. The right decision at the right time regarding implementing a smart emergency level will be beneficial for the small/medium enterprises (SMEs), and the economy of the state. The study is a form of the disaster management approach for the traders, logistics, retailers, manufacturers, and supply chains to manage resources and production optimally to deal successfully with unbalanced markets during the COVID-19 pandemic.

The proposed research model can be extended to a three-echelon SCM by including retailer or wholesellers. The reason is that these small enterprises are more affected by emergencies (social distancing and lockdown) created by the COVID-19 pandemic because of their asset limitations and for this purpose a rapid, timely and decisive plan is required. The demand variation during a pandemic may be decreased by following certain other distributions. The essence of randomization using stochastic modeling can be incorporated to make the proposed model more generic and realistic. Further, the proposed model can also be expanded in order to consider more realistic scenarios such as multi-echelon supply chains with single-buyer multiple vendors, multiple buyers and single vendors or multiple buyers and multiple vendors. Overall, the COVID-19 pandemic is dangerous for the world economy due to the resulting short term and long term global SCM disruption. However, in the current scenario, industries need to face this challenge with timely proactive approaches to avoid irreparable losses.

## Figures and Tables

**Table 1:** Previous author relevant contributions in the research.

Author(s)	Model Type		Production Rate		Production Parameter		Imperfect Production	Resource Leveling		Cost Minimization	Disaster Based Model		Environment	
	SCM	Production System	Constant	Variable	Lead Time	Cycle Time		Workforce	Machines		Geo- Thermal	Pandemic	Deterministic	Uncertain
Sabegh (2017) [10]	√	√	√		√			√		√	√		√	
Song (2018) [11]	√			√							√			√
Cao (2018) [12]	√							√		√	√		√	
Kaur and Singh (2019) [13]	√	√		√	√				√	√	√		√	
Mishra (2017) [18]	√										√			
Ouyang (2002) [27]		√	√		√		√			√				
Jha (2009) [28]	√		√		√					√				
Yi (2013) [31]	√		√		√									
Glock (2011) [37]		√		√					√	√				
Sarkar (2018) [41]	√		√		√		√			√				
Connolly (2002) [47]		√	√											
Proposed Re- Search	√	√		√		√	√	√	√	√		√		√

**Table 2:** The emergency levels with status and real-life conditions from 0 to 5.

Emergency Level (Ω)	Emergency Status	Conditions
0	No emergency	No pandemic, where the conditions are normal and favorable for production and sales.
1	Preventive level	Social distancing; Educational sectors and social gathering closed, checking and testing of workforce internationally.
2	Preliminary lockdown	Unessential commodity retailers closed, Inter-professional/states public transportation closed, workforce disturbance, partial lockdown of lavish commodity retailers.
3	Partial lockdown	Complete closure of public transport, inter-district transports, and unessential commodity retailers, supply shortages, demand supply imparity.
4	Partial curfew	Time slabs allotted to important personality and needy people for movements, severe supply shortages to the retailers and manufacturers.
5	Fully curfew	Complete curfew and full lockdown, no movements are allowed except serious emergencies, only authorized transport available for supply to retailer/manufacturer.

**Table 3:** Automobile industry manufacturer data.

Product Type	Tool-Die Cost ($/machine)	Fixed Production Cost ($/unit)	Holding ($/unit/year)	Raw Material ($/unit)	Setup ($/year)	Labor ($/labor-year)	Demand (units/year)
A	0.01	600	0.5	18	10	1000	900
B	0.01	610	0.5	19.5	1	1000	800
C	0.02	628	0.5	21	12	1000	800

**Table 4:** Automobile industry vendors’ data.

Product Type	Tool-Die Cost($/machine)	Fixed Prod. Cost ($/unit)	Holding ($/unit/year)	Raw Material (%)	Fixed Inspect. ($/year)	Variable Inspection ($/unit)	Rework ($/unit)	Disposal ($/unit/year)
A	0.01	600	0.5	18	5	0.1	0.116	0.83
B	0.01	610	0.5	19.5	5.5	0.2	0.116	0.83
C	0.02	628	0.5	21	6	0.3	0.15	0.83

**Table 5:** The optimal result of the supply chain model with emergency level Ω = 1.

Decision Variable	Abbreviation	SQP (Exponential Demand)	SQP (Linear Demand)	Pattern Search (Linear Demand)	GA (Linear Demand)
Cycle times	T1	0.0383	0.022	0.022	0.022
	T2	0.0424	0.025	0.025	0.025
	T3	0.0419	0.025	0.025	0.025
Machine units	Kja1	4	6	6	6
	Kja2	3	5	5	5
	Kja3	3	5	5	5
	Kjc1	4	7	7	7
	Kjc2	4	6	6	74
	Kjc3	4	6	6	6
Labor required	Lja1	10	15	15	15
	Lja2	7	12	12	12
	Lja3	7	12	12	12
	Ljc1	10	17	17	17
	Ljc2	10	15	15	15
	Ljc3	10	15	15	15
Plant production	Pja1	600	900	1350	1350
	Pja2	480	800	800	800
	Pja3	510	850	850	850
	Pjc1	520	910	910	910
	Pjc2	560	840	840	840
	Pjc3	600	900	900	900
Total cost of SCM	TCj	124,835.5	226,282.3	226,282.3	507,138.85

**Table 6:** The utilization of resources with respect to the emergency levels (Ω) due to COVID-19 pandemic.

Decision Variables	Abbreviations	Ω = 0	Ω = 1	Ω = 2	Ω = 3	Ω = 4	Ω = 5
Machines utilization	K1a	7	6	6	5	4	4
	K2a	6	5	5	4	4	3
	K3a	6	5	5	4	3	3
	K1c	8	7	7	6	5	4
	K2c	7	6	5	5	4	3
	K3c	6	6	5	4	4	3
	L1a	17	15	15	12	10	10
	L2a	15	12	12	10	10	7
	L3a	15	12	12	10	7	7
Labors required	L1c	19	17	17	15	12	10
	L2c	17	15	12	12	10	7
	L3c	15	15	12	10	10	7
	P1a	1050	900	900	750	600	600
	P2a	960	800	800	640	640	480
	P3a	1020	850	850	680	510	510
Plant Production rate	P1c	1040	910	910	780	650	520
	P2c	980	840	700	700	560	420
	P3c	900	900	750	600	600	450
Total cost of SCM	TCj	263,420.5	226,282	201,373	166,475	137,547	110,251

## Data Availability

The data presented in this study are available on request from the corresponding author. The data are not publicly available due to large data sets.
